# Floral Mass per Area and Water Maintenance Traits Are Correlated with Floral Longevity in *Paphiopedilum* (Orchidaceae)

**DOI:** 10.3389/fpls.2017.00501

**Published:** 2017-04-07

**Authors:** Feng-Ping Zhang, Ying-Jie Yang, Qiu-Yun Yang, Wei Zhang, Tim J. Brodribb, Guang-You Hao, Hong Hu, Shi-Bao Zhang

**Affiliations:** ^1^Key Laboratory of Economic Plants and Biotechnology, Kunming Institute of Botany – Chinese Academy of SciencesKunming, China; ^2^Yunnan Key Laboratory for Research and Development of Wild Plant Resources, Kunming Institute of Botany – Chinese Academy of SciencesKunming, China; ^3^Yunnan Key Laboratory of Flower BreedingKunming, China; ^4^University of Chinese Academy of SciencesBeijing, China; ^5^Department of Plant Sciences, University of Tasmania, HobartTAS, Australia; ^6^Institute of Applied Ecology of Chinese Academy of SciencesShenyang, China

**Keywords:** floral longevity, floral dry mass per unit area, floral economic traits, floral water maintenance, *Paphiopedilum*

## Abstract

Floral longevity (FL) determines the balance between pollination success and flower maintenance. While a longer floral duration enhances the ability of plants to attract pollinators, it can be detrimental if it negatively affects overall plant fitness. Longer-lived leaves display a positive correlation with their dry mass per unit area, which influences leaf construction costs and physiological functions. However, little is known about the association among FL and floral dry mass per unit area (FMA) and water maintenance traits. We investigated whether increased FL might incur similar costs. Our assessment of 11 species of *Paphiopedilum* (slipper orchids) considered the impact of FMA and flower water-maintenance characteristics on FL. We found a positive relationship between FL and FMA. Floral longevity showed significant correlations with osmotic potential at the turgor loss and bulk modulus of elasticity but not with FA. Neither the size nor the mass per area was correlated between leaves and flowers, indicating that flower and leaf economic traits evolved independently. Therefore, our findings demonstrate a clear relationship between FL and the capacity to maintain water status in the flower. These economic constraints also indicate that extending the flower life span can have a high physiological cost in *Paphiopedilum*.

## Introduction

Floral longevity (FL), defined as the length of time that a flower remains open and functional, influences the processes of pollen removal and pollination (Primack, 1985; Ashman and Schoen, 1994). This functional trait varies greatly among species and is an important contributor to increased reproductive success because a longer flowering period can allow plants to attract more pollinators. However, a trade-off may exist between the benefit of increased pollination success and the cost of floral maintenance (Rathcke, 2003). Researchers have suggest that FL is affected by many biotic (Rathcke, 2003; Giblin, 2005; Weber and Goodwillie, 2012) and abiotic factors (Vespirini and Pacini, 2005; Arroyo et al., 2013; Jorgensen and Arathi, 2013). For example, longer flower life spans might be associated with less frequent pollination (Giblin, 2005; Weber and Goodwillie, 2012). Longevity is also improved in plants growing at higher elevations or lower temperatures, or in areas where soil moisture is high (Vespirini and Pacini, 2005; Arroyo et al., 2013; Jorgensen and Arathi, 2013).

Leaf dry mass per unit area (LMA) is a central trait in ecology (Poorter et al., 2009), which shows strong correlations with a suite of important leaf functional traits across a diverse group of species (Small, 1972; Reich et al., 1997; Wright et al., 2004). Longer lived leaves have higher LMA, which influences leaf construction cost and physiological functions such as water transport and use (Fu et al., 2012). A high LMA is typical for stress tolerant species (Wright et al., 2004; Poorter et al., 2009). A direct consequence of greater LMA in leaves with higher lifespan is reduced photosynthetic rate per mass investment. High LMA is thus typically associated with greater drought tolerance (Wright et al., 2004; Poorter et al., 2009). Unlike leaves, flowers do not contribute much to carbon assimilation and are relatively short lived but still may transpire significant amounts of water (Roddy and Dawson, 2012; Teixido and Valladares, 2014). On the contrary, they often experience desiccating conditions that would lead to wilting and prevent pollen dispersal. Therefore, they must maintain water balance and turgor during flowering to attract pollinators. Although longer-lived flowers may increase the opportunities for pollination and reproductive success, especially in habitats where pollinators are in shorter supply, however the attendant costs to those plants are uncertain. This phenomenon of life span versus construction costs has already been described for leaves. In fact, the “leaf economic spectrum” occurs across all major terrestrial plant groups, where a higher (LMA) is invested in longer-lived leaves (Wright et al., 2004; Sack et al., 2013; John et al., 2017). The role of water transport traits in flower evolution is complex, with phylogeny an important determinant of flower hydraulic characteristics (e.g., vein density) that has evolved independently of leaves (Roddy et al., 2013). In addition, the benefits associated with longevity are also very different in flowers when compared to leaves. Thus, it is important to understand whether flowers exhibit trait linkages with longevity similar to those found for leaves.

An adequate water supply is needed during all periods of floral display, including bud expansion, flower opening, nectar production, and the maintenance of turgor in floral organs (Mohan Ram and Rao, 1984; Patino and Grace, 2002; Tsukaguchi et al., 2003; van Doorn and van Meeteren, 2003; Galen, 2005). Flower maintenance may require a considerable amount of water (Roddy and Dawson, 2012) and, under dry conditions, those plants can lose more water from their flowers than from the leaves (Lambrecht, 2013). Therefore, understanding the physiological mechanisms of floral water transport and water relations may provide new insights into the evolution of flowers (Galen, 2000; Chapotin et al., 2003; Feild et al., 2009a,b; Roddy et al., 2016). Even though water has an essential role throughout the floral lifespan, few studies have focused on water relations in flowers (Feild et al., 2009b; Lambrecht et al., 2011; Roddy et al., 2016). Contrasting results in different species have indicated that some flowers are phloem-hydrated (Trolinder et al., 1993; Chapotin et al., 2003). Other studies, such as that involving *Magnolia grandiflora*, has shown that the giant flowers of that species are hydrated by the xylem (Feild et al., 2009a,b). However, quantitative investigations have been lacking about water relations in flowers from different species, even though they can vary greatly in floral characteristics such as longevity, size, color, and shape.

Orchids, an important group of plants both economically and ecologically, are well-known for their ornamental flowers. In addition, many species show enhanced drought tolerance due to their epiphytic growth habit (Zhang et al., 2012). The genus *Paphiopedilum* exhibits wide variations in floral life span (from 15 to 60 days) as well as high diversity in morphology and physiology (Karasawa and Saito, 1982; Guan et al., 2011; Zhang et al., 2011). This makes it an ideal system for studying potential functional associations between FL and flower physiology. Moreover, this genus has a well-studied phylogeny that can facilitate phylogenetically based data analyses (Cox et al., 1997) and the interpretation of patterns of functional trait evolution.

Here, we examined FL, floral dry mass per unit area (FMA), and pressure–volume traits using the flowers from 11 *Paphiopedilum* species growing in southwestern China. Our aim was to identify important functional associations between flower life span and water relations. Specifically, we hypothesized that, similar to leaves, flowers exhibit a positive correlation between cost (in terms of mass per unit area) and longevity, such that flowers from species with greater FL would also have higher FMA values. We also examined whether floral traits are correlated with leaf traits or they are independent of each other due to different selective pressures they experienced.

## Materials and Methods

### Study Site and Plant Materials

This year-long examination was conducted in 2013 at the Kunming Institute of Botany – Chinese Academy of Sciences (25°08′ N, 102°44′ E, elevation 1912 m), in southwestern China. 11 studied species *Paphiopedilum* were collected in the wild and then cultivated in the greenhouse under conditions that included 30–40% full sunlight (controlled by shade nets) and ambient temperatures of 20–25°C, the *Paphiopedilum* species grown well under such optimal conditions. The broken bark medium was used to grow the collected plants, and they were watered as needed (1–2 times per week). These plants were growing in the same greenhouse for 5 years and differences due to varying ambient environment factors experienced in the wild are thus minimized.

### Measurements of Floral and Leaf Functional Traits

**FIGURE 1:**
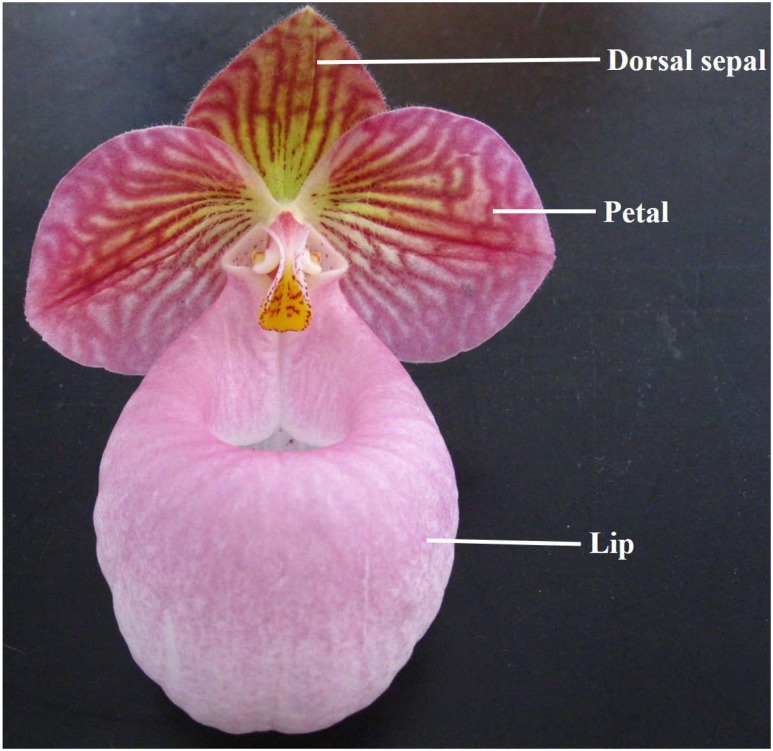
**Typical floral structure of *Paphiopedilum* (here depicted by *P*. *micranthum*)**.

Floral and leaf functional traits were evaluated at the peak flowering time for each species. A flower was regarded as “opening” when the dorsal sepal rose, and remained functional. Six leaves and flowers per species were excised in the morning, and then sealed in plastic bags and immediately transported to our nearby laboratory. The floral area (FA) of all organs in a flower, i.e., dorsal sepal, petal, and lip (**Figure 1**), and leaf area (LA) were determined with a Li-Cor 3000A area meter (Li-Cor, Inc., Lincoln, NE, USA). Afterward, these flowers and leaves were oven-dried at 70°C for 48 h to obtain their dry weights (DW). FMA (g m^-2^) was calculated as DW/FA and LMA (g m^-2^) was calculated as DW/LA.

### Flower Pressure–Volume Curve

**FIGURE 2:**
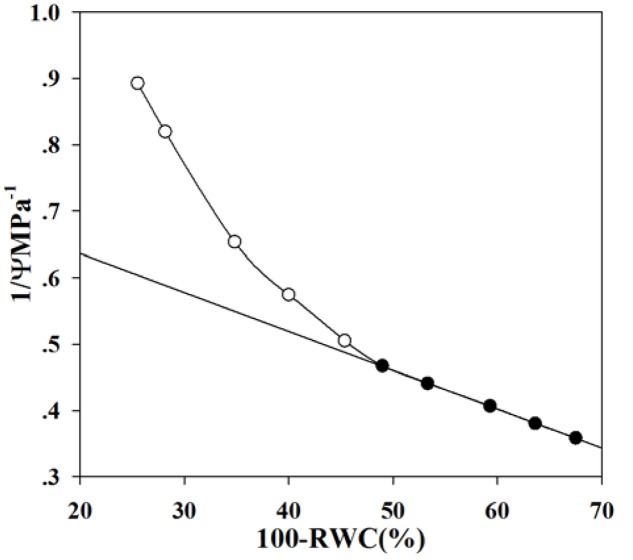
**The typical pressure–volume curve of *Paphiopedilum* (here depicted by *P*. *malipoense*)**. Low values of both water potential and RWC were obtained from the very start of the experiment because water potential was measured with a WP4 Dewpoint Potentiometer, which determines the relative humidity of the air above a flower sample in a closed chamber. Thus, species flower turgor loss points are likely more negative than if flower water potential had been measured with a pressure chamber.

Mature flowers were quickly sampled from five or six plants per species early in the morning, sealed in plastic bags, and immediately transported to the laboratory. After the scapes were re-cut under water, these flowers were soaked in deionized water for 12 h to achieve full hydration, and they were weighed to obtain their saturated fresh weights (FW_s_). The samples were then cut into segments in a plastic bag with damp paper towel to prevent dehydration by transpiration in air and rapidly placed in individual chambers (diameter 3.7 cm) for the WP4 Dewpoint Potentiometer (Decagon Devices, Inc., Pullman, WA, USA). After equilibration for approximately 30 s, the flower water potentials were recorded before FW_s_ were measured to the nearest 0.0001 *g* on a digital balance. Water potentials and FW were determined periodically until those values stabilized. The samples were then oven-dried at 70°C for 24 h to obtain DW, and relative water content (RWC) was calculated as (FW-DW)/(FW_s_-DW). Pressure–volume curves (**Figure 2**) were obtained by plotting the inverse of water potential against RWC. The WP4 Dewpoint Potentiometer measures water potential by determining the relative humidity of the air above a sample in a closed chamber, thus the inability to get more hydrated values, so that the first point has low water potential and RWC (**Figure 2**). Turgor loss point was determined as the point of transition between linear and non-linear portions of the curve. Osmotic potential at the turgor loss point (π_tlp_) and relative water content at this point (RWC_tlp_) were also recorded accordingly (Tyree and Hammel, 1972). Osmotic potential at full turgor (π_ft_) was estimated by extrapolating the linear portion of the curve to 100% RWC, and relative water content at full turgor (RWC_ft_) was estimated by extrapolating the line to zero osmotic potential. Then the bulk modulus of elasticity (ε) was calculated as (π_ft_-π_tlp_) × (RWC_ft_ – RWC_tlp_)/RWC_ft_.

### Floral Longevity

To investigate the FL of a single flower from each species of *Paphiopedilum*, we sampled 10–20 plants and randomly marked 10–20 newly emerged floral buds per species. Their individual opening and wilting dates were recorded throughout the flowering season. Each floral bud was sampled from a separate plant. A flower was identified as “opening” when the dorsal sepal rose and any visiting pollinator could enter the pouched labellum. The flower was regarded as “wilting” when the labellum began to droop, thereby ending its role in the pollination process.

### Data Analysis

All analyses were performed using the R software program (version 2.15.0; R Development Core Team, 2012). Relationships among traits were examined by Pearson’s correlations (cor. test function in the R package). Our phylogenetic tree of *Paphiopedilum* species was constructed according to the method described by Cox et al. (1997). Phylogenetically independent contrasts (PICs; Felsenstein, 1985) were calculated by applying the ‘pic’ function in the package *picante* for the R software.

## Results

**FIGURE 3:**
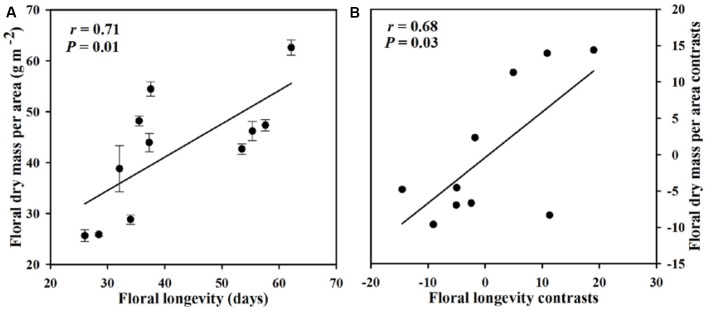
**Pearson correlations (A)** and phylogenetically independent contrast correlations **(B)** of floral longevity (FL) with floral dry mass per unit area (FMA) across 11 *Paphiopedilum* species.

**FIGURE 4:**
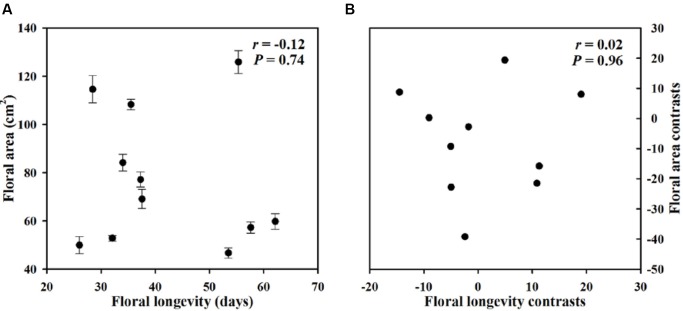
**Pearson correlations (A)** and phylogenetically independent contrast correlation **(B)** of FL with floral area (FA) across 11 *Paphiopedilum* species.

**FIGURE 5:**
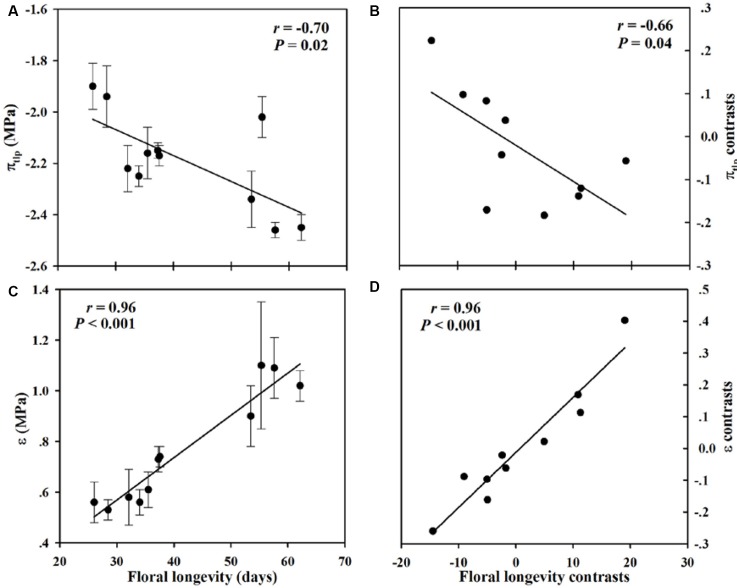
**Pearson correlations (A,B)** and phylogenetically independent contrast correlations **(C,D)** of FL with turgor loss point (π_tlp_) and modulus of elasticity (ε) across 11 *Paphiopedilum* species.

Within our sample group of 11 species of *Paphiopedilum*, we found large interspecific diversity in leaf dry mass per unit area, leaf area, flower dry mass per unit area, floral area, FL, turgor loss point, and bulk modulus of elasticity (**Table 1**). Furthermore, significant relationships were found among traits associated with FL, FMA, and flower maintenance. Whereas, FL was positively correlated with FMA (*P* = 0.01), but not with FA (*P* = 0.74) (**Figures 3**, **4**). Even after phylogeny was considered, FL was still correlated with FMA (*P* = 0.03) (**Figure 3**). Longevity was correlated negatively with π_tlp_ (*P* = 0.02) and positively with ε (*P* < 0.001) (**Figure 5**). These correlations of FL with π_tlp_ and ε remained significant before and after accounting for the phylogenetic relationships (π_tlp_ : *P* = 0.04), ε: *P* < 0.001).

**Table 1:** Quantification of floral and leaf functional traits for selected *Paphiopedilum* species.

Species	LMA (g m^-2^)	LA (cm^2^)	FMA (g m^-2^)	FA (cm^2^)	FL (Days)	π_tlp_ (MPa)	ε (MPa)
*P*. *appletonianum*	69.24 2.25	42.94 ± 6.12	42.69 ± 1.02	46.72 ± 2.10	53.47 ± 1.31	-2.34 ± 0.11	0.90 ± 0.12
*P*. *armeniacum*	112.80 5.15	16.63 ± 0.65	28.83 ± 0.91	84.22 ± 3.51	34.00 ± 0.63	-2.25 ± 0.04	0.56 ± 0.05
*P*. *charlesworthii*	78.07 4.22	13.99 ± 0.95	25.64 ± 1.15	49.94 ± 3.59	26.00 ± 0.74	-1.90 ± 0.09	0.56 ± 0.08
*P*. *dianthum*	166.45 5.30	92.99 ± 8.35	62.61 ± 1.47	59.77 ± 3.29	62.13 ± 1.10	-2.45 ± 0.05	1.02 ± 0.06
*P*. *henryanum*	92.84 5.21	22.37 ± 2.33	38.81 ± 4.57	52.83 ± 1.14	32.07 ± 0.66	-2.22 ± 0.09	0.58 ± 0.11
*P*. *hirsutissimum*	128.07 4.84	51.82 ± 4.41	54.46 ± 1.40	69.06 ± 3.96	37.53 ± 0.51	-2.17 ± 0.04	0.74 ± 0.04
*P*. *malipoense*	115.64 7.39	65.61 ± 5.41	46.20 ± 1.87	125.92 ± 4.78	55.33 ± 1.09	-2.02 ± 0.08	1.10 ± 0.25
*P*. *micranthum*	166.51 14.92	20.29 ± 1.23	25.86 ± 0.33	114.58 ± 5.68	28.45 ± 0.55	-1.94 ± 0.12	0.53 ± 0.04
*P*. *tigrinum*	99.30 6.88	39.59 ± 1.81	43.96 ± 1.79	77.16 ± 3.19	37.27 ± 0.67	-2.15 ± 0.03	0.73 ± 0.05
*P*. *villosum*	118.20 4.01	35.22 ± 1.94	48.21 ± 0.94	108.30 ± 2.20	35.50 ± 0.81	-2.16 ± 0.10	0.61 ± 0.07
*P*. *wardii*	72.49 6.50	31.95 ± 3.57	47.38 ± 1.11	57.28 ± 2.30	57.60 ± 0.49	-2.46 ± 0.03	1.09 ± 0.12

*LMA, leaf dry mass per unit area; LA, leaf area; FMA, flower dry mass per unit area; FA, floral area; FL, floral longevity; π_tlp_, floral turgor loss point; ε, floral bulk modulus of elasticity*.

**FIGURE 6:**
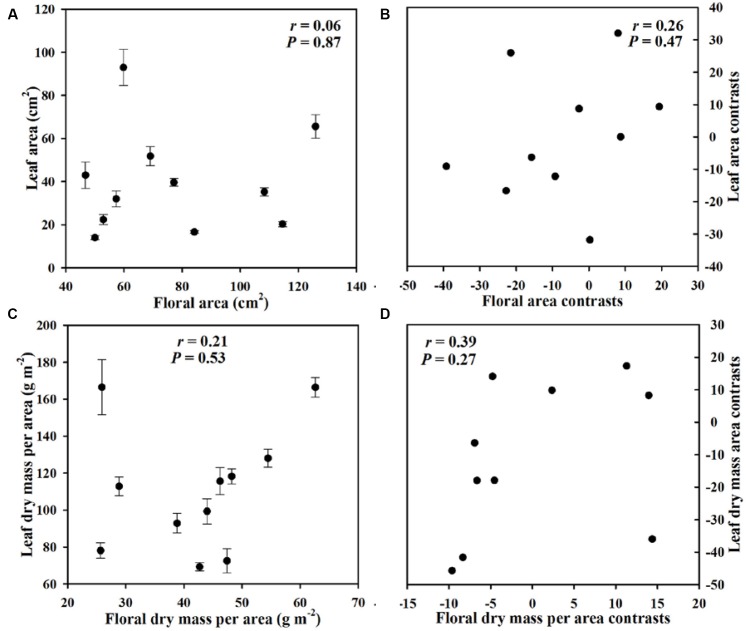
**Pearson correlations (A,B)** and phylogenetically independent contrast correlations **(C,D)** of FA with leaf area (LA) and of leaf dry mass per unit area (LMA) with FMA across 11 *Paphiopedilum* species.

We observed no correlation between FA and LA (*P* = 0.87), and FA was still not correlated with LA after considering phylogeny (*P* = 0.47) (**Figure 6**). Likewise, phenotypically and phylogenetically, FMA showed no relationship with LMA (Pearson correlation: *P* = 0.53, phylogenetically independent contrast correlation: *P* = 0.27) (**Figure 6**).

## Discussion

Our results solidly support the hypothesis that FL is tightly coupled to the cost of flower maintenance in terms of flower dry mass per unit area and drought tolerance. This study provides novel insight into the physiological mechanism for maintaining FL in species of *Paphiopedilum*, and our data reveal important functional associations related to the maintenance of longer-lived flowers based on water relations in *Paphiopedilum*.

### Correlation of Floral Longevity with Water Availability and Maintenance

These results strongly indicate that longevity is evolutionarily correlated with flower dry mass per unit area (**Figure 3**). This relationship seems to mirror the correlation reported between leaf dry mass per unit area and leaf life span, with LMA being a key functional trait in plant performance (Lambers and Poorter, 1992) as well as a critical indicator of plant adaptive strategies (Poorter et al., 2009). For example, leaves from species with higher LMA tend to have longer life spans (Ryser, 1996; Poorter et al., 2009) and, consequently, those plants conserve acquired nutrients and carbon more efficiently. Our data present a surprisingly strong variation in FMA among species. Those with longer-lived flowers show higher FMA values than species with shorter-lived flowers. The high-FMA species also have greater floral density, which is expected because FMA is the product of thickness and density. This high-FMA trait might also enhance the residence time of water in plants, thus providing those particular species with a competitive advantage when moisture is less available. Therefore, these correlations suggest that, similar to the influence of LMA, FMA is the nexus for a suite of functional traits.

We also found that FL is closely associated with the capacity to maintain flower turgor. This was shown by our Pearson’s and PICs analyses, which demonstrated that longer-lived flowers are more capable of maintaining turgor. Such flowers also have lower values for π_tlp_ and higher values for ε, implying that they are more tolerant of drought stress (Bartlett et al., 2012). The turgor loss point reported is likely more negative than what it should be because it was measured with a dewpoint potentiometer which gives lower values than with the pressure chamber. Evolutionary correlations have previously been reported between leaf life span and leaf water potential at the turgor-loss point (Fu et al., 2012). Our new data allow us to extend this conclusion to the flowers in *Paphiopedilum*. A more negative π_tlp_ and a more positive ε broadens the range of values for leaf water potential at which plant tissues remain turgid and maintain their functions (Sack et al., 2003; Lenz et al., 2006; Bartlett et al., 2012). Therefore, all of these findings point to a critical application of π_tlp_ and ε as key functional traits for maintaining floral and leaf longevity.

### Correlations between Flower and Leaf Traits

For the *Paphiopedilum* species investigated here, leaf and flower sizes are not correlated with mass/area (**Figure 6**), which strongly suggests that orchid flower and leaf economic traits have evolved independently. This physiological modularity makes good sense in the context of differing selective pressures upon non-photosynthetic petals versus highly photosynthetic leaves. Studies of variations in morphological and physiological traits have found similar results and have emphasized a genetic basis for reproductive and vegetative modularity (Juenger et al., 2005; Pélabon et al., 2011). Although inflorescence size appears to be coordinated with leaf size in the Proteaceae (Midgley and Bond, 1989), that relationship may not extend to individual flowers. In *Dalechampia scandens*, floral bract length is more relevant to variations in pollination-related floral traits than to variations in leaf traits (Pélabon et al., 2011). For *Arabidopsis thaliana*, quantitative trait loci mapping of leaf- and flower-size traits has not revealed any correlations between those trait categories (Juenger et al., 2005). Furthermore, Roddy et al. (2013) found no proof of correlated evolution for leaf and petal venation patterns across the angiosperm phylogeny. Those earlier results, as well as our data, suggest that the physiological traits of leaves and flowers may arise from non-correlated selection pressures and functions.

## Conclusion

Our findings support the hypothesis that FMA is positively correlated with FL, and they provide strong evidence that drought tolerance coincides with FL in *Paphiopedilum*. Species with different flower life spans also vary in their flower water relations. These contrasting strategies among species are vital to their survival because different capacities for water maintenance must mean different costs. Although researchers are beginning to explore these processes in vegetative organs, such as leaves and stems (Fu et al., 2012; Simonin et al., 2012; Carins-Murphy et al., 2014), little attention has been given to their significance in reproductive organs. Further investigations into the costs of sustaining FL in orchids under a range of ecological conditions and amid various growth forms are required if we are to understand how this lineage of species has become so successful.

## Author Contributions

F-PZ, S-BZ, and HH designed this study; F-PZ, Y-JY, Q-YY, and WZ conducted the experiments; F-PZ performed the analysis and wrote the manuscript; and TB and G-YH helped to evaluate and edit the manuscript.

## Conflict of Interest Statement

The authors declare that the research was conducted in the absence of any commercial or financial relationships that could be construed as a potential conflict of interest.
